# Modeling sex differences in Alzheimer’s Disease using isogenic hiPSC lines with different sex chromosome complements and APOE alleles

**DOI:** 10.1002/alz.093548

**Published:** 2025-01-03

**Authors:** Gala N Filippova, Michelle Casad, Camille Groneck, Katherine Hui, Swati Mishra, James W MacDonald, Theo Bammler, Daniel L Van Dyke, Anna Skakkebaek, Claus H Gravholt, Julie Mathieu, Xinxian Deng, Joel B Berletch, Suman Jayadev, Christine M Disteche, Jessica E. Young

**Affiliations:** ^1^ University of Washington, Seattle, WA USA; ^2^ Institute for Stem Cell and Regenerative Medicine, Seattle, WA USA; ^3^ Mayo Clinic, Rochester, MN USA; ^4^ Aarhus University, Aarhus Denmark

## Abstract

**Background:**

Late Onset Alzheimer’s Disease (LOAD) is the most common neurodegenerative disorder. Carriers of an ɛ4 allele of the apolipoprotein E gene (APOE) have significantly increased risk of developing LOAD. LOAD is also strongly sex biased. Besides the well‐established role of sex hormones, the impact of genetic sex combined with APOE genotypes has not been studied. Sex chromosome dosage (SCD) is the main genetic difference between females (XX) and males (XY). Despite dosage compensation by X chromosome inactivation (XCI), some X‐linked genes remain expressed from the inactive X (Xi) and are female biased in expression. Many of these genes are related to neurological and immune functions and could contribute to neuroinflammation in AD.

**Method:**

To understand the genetic interactions between autosomal risk (APOE) and sex‐bias in AD, we are generating isogenic sets of hiPSCs with various sex chromosome complements and APOE genotypes. We have derived XXY/XY, XY/X, and XX/X isogenic pairs by reprogramming cells from naturally mosaic individuals or by selective removal of the Xi *in vitro.* These lines uniquely position us to differentiate the effects of X chromosome dosage and/or presence of a Y chromosome, while minimizing genetic variability and environmental or hormonal confounders. Currently, we are also constructing APOE ɛ3 and ɛ4 alleles in our paired hiPSC lines with differing sex chromosome content using gene editing.

**Result:**

Starting from an XXY/XY isogenic pair, we have generated an hiPSC series with six different genotypes of APOE ɛ3/ɛ3, ɛ3/ɛ4 or ɛ4/ɛ4 alleles with either the XXY or XY genotype. Preliminary results in differentiated neural precursor cells show differential expression of sex‐linked genes in relation to SCD. We also observed genome‐wide changes in gene expression and DNA methylation. Subsets of autosomal sex‐biased genes were differentially affected either by X chromosome dosage, presence of a Y, or both.

**Conclusion:**

The new hiPSC lines are being differentiated to AD‐relevant cell types, including neurons, microglia, and cortical organoids, for transcriptomic and functional analyses to elucidate how APOE alleles and genetic sex interact to modulate risk in LOAD pathology.

